# Comparing pharmacologic measures of tenofovir exposure in a U.S. pre-exposure prophylaxis randomized trial

**DOI:** 10.1371/journal.pone.0190118

**Published:** 2018-01-09

**Authors:** Sanjiv M. Baxi, Eric Vittinghoff, Peter Bacchetti, Yong Huang, Kata Chillag, Ryan Wiegand, Peter L. Anderson, Robert Grant, Ruth M. Greenblatt, Susan Buchbinder, Monica Gandhi, Albert Y. Liu

**Affiliations:** 1 Department of Medicine, University of California, San Francisco, California, United States of America; 2 School of Public Health, Division of Epidemiology, University of California, Berkeley, California, United States of America; 3 Department of Epidemiology and Biostatistics, University of California, San Francisco, California, United States of America; 4 Department of Bioengineering and Therapeutic Sciences, University of California, San Francisco, California, United States of America; 5 Division of HIV/AIDS Prevention, National Center for HIV, Viral Hepatitis, STD and TB Prevention, Atlanta, Georgia, United States of America; 6 Division of Parasitic Diseases and Malaria, U.S. Centers for Disease Control and Prevention, Atlanta, Georgia, United States of America; 7 Skaggs School of Pharmacy and Pharmaceutical Sciences, University of Colorado, Aurora, Colorado, United States of America; 8 Gladstone Institutes, San Francisco, California, United States of America; 9 San Francisco AIDS Foundation, San Francisco, California, United States of America; 10 Department of Clinical Pharmacy, University of California, San Francisco, California, United States of America; 11 San Francisco Department of Public Health, San Francisco, California, United States of America; Imperial College London, UNITED KINGDOM

## Abstract

It is critical to assess adherence to pre-exposure prophylaxis (PrEP) in clinical trials. The relationship between pharmacokinetic measures of PrEP adherence and other adherence measures used in PrEP trials requires further characterization. Plasma, peripheral blood mononuclear cells (PBMCs), and hair samples were collected from 88 HIV-uninfected men who have sex with men in a placebo-controlled randomized PrEP trial; announced pill counts, medication electronic monitoring (MEMS), and self-report using visual analog scale (VAS) were also collected. Tenofovir (TFV or TFV-DP) in plasma, hair, and PBMCs were quantified. Proportions with drug detection, Spearman correlation coefficients, and univariate and multivariable regression models were used. Drug detection in plasma, PBMC, and hair samples was highly concordant with treatment arm assignment. TFV or TFV-DP levels were detected in most active-arm participants: 44/47 (94%) in plasma, 46/47 (98%) in hair, and 44/47 (94%) in PBMCs and in only 1/41 placebo arm participant. Correlation coefficients were r = 0.59 for plasma and PBMC, r = 0.34 for PBMC and hair and r = 0.36 for plasma and hair. MEMS and announced pill-counts were moderately correlated (r = 0.52) but less so with pharmacologic measures (range of r = 0.12 to 0.38). Self-reported adherence by visual analog scale demonstrated essentially no correlation with drug levels (r = 0.06 for hair, PBMC or plasma) and was a poor indicator of exposure to study product. Indices of drug exposure are important indicators in assessing adherence to PrEP; accounting for variability between measures may improve interpretation and use of adherence measures in future PrEP studies.

**Clinical trial registration:**

Clinical Trials.gov NCT00131677

## Introduction

Several HIV pre-exposure prophylaxis (PrEP) clinical trials have demonstrated the safety and efficacy of daily oral tenofovir disoproxil fumarate (TDF) either alone or in combination with emtricitabine (FTC) in the prevention of HIV infection [1–6]. As a result of these studies, both the US Centers for Disease Control and Prevention (CDC) [7] and the World Health Organization (WHO) [8] have published guidelines for PrEP use. Adherence to a daily PrEP regimen is the most important determinant of PrEP effectiveness. Recent PrEP trials have demonstrated that adequate adherence to PrEP is imperative for benefit and that self-reported adherence and pill counts have limited utility in assessing adherence [9–12]. Therefore, adequate measurement of PrEP use was essential to interpret clinical trial outcomes, in particular those showing limited benefit, and ultimately in determining what amount of PrEP use is sufficient for effective prevention of HIV acquisition.

Several different measures of adherence to study product have been used with varying success in HIV PrEP trials, but each has its limitations. Self-report of adherence is influenced by social-desirability bias, recall bias and poor memory [13–15]. Scheduled or unannounced pill counts by study staff or medication event monitoring system (MEMS) recording of bottle cap-openings may represent an improvement over self-report [16, 17], but are subject to end-user variability and fail to account for tampering, decanting or sharing of medications. Moreover, both pill counts and MEMS fail to confirm actual pill ingestion [13, 18–23]. For these reasons, there has been interest in the implementation of biological or pharmacologic measures of drug exposure in PrEP trials, where concentrations of the drug of interest are measured in a biologic matrix.

Antiretroviral (ARV) measurement in plasma has been frequently used in PrEP trials, and reflects whether a recent dose was ingested, but does not inform cumulative dosing beyond the several days prior to the specimen collection [24–26]. Furthermore, plasma levels are susceptible to “white-coat” consumption of study drug in anticipation of clinic visits, in which case the patient would appear to be adherent even if non-adherent prior to the day or days preceding the study visit [27, 28]. Drug levels in PBMCs and red blood cells measured in dried blood spots (DBS) have also proven useful in the interpretation of PrEP trial results [1, 11, 29, 30] by providing information on exposure over longer periods (7–14 days for PBMC, 30 days for DBS) and because they are strongly correlated with PrEP efficacy [11, 29, 30]. However, procedures to process, isolate and count PBMCs can be technically challenging and expensive. DBS collection and processing is simpler, although freezing is required to preserve the medication moieties that are indicative of long-term PrEP exposure. Hair collection is noninvasive, hair specimens can be stored and shipped at room temperature, and ARV levels in hair reflect uptake from the systemic circulation over weeks-to-months [31], thus capturing long-term cumulative exposure. While the concentration of ARVs in hair does not provide information on recent variability in patterns of exposure, nor on the time course of variable exposure, hair is easy to handle and store, has been highly acceptable in a variety of studies [15], offering possible advantages in resource-limited settings.

Several gaps remain in our understanding of these three pharmacologic measures of PrEP adherence. First, we need to know which can accurately distinguish PrEP use from non-use with various “look-backs” in time. Second, we need to understand what factors influence drug levels. Finally, we need to understand how well various pharmacokinetic (PK) measures correlate with each other and with traditional measures of adherence. Having this information will aid in interpretation of PK measures of adherence in future PrEP projects and roll-out settings. We report findings of a nested substudy within the US CDC PrEP Safety Study, which provided a unique opportunity to compare the utility of hair, PBMC, and plasma measures in detecting study product use in a placebo-controlled, randomized, PrEP study design. We also estimated the correlations between drug concentrations in hair, PBMC and plasma samples, as well as their correlations with MEMS caps openings, announced pill counts, and self-report by visual analog scale, and finally assessed the impact of factors that may influence these drug levels, including age, weight, creatinine clearance, and hair color and treatment.

## Materials and methods

### Study design, sample collection and participants

The US CDC Safety Study was a multi-site phase 2, randomized, double-blind (investigator and participant), placebo-controlled extended-safety trial of TDF among MSM in the United States (Clinical Trials.gov NCT00131677). This multi-site study enrolled 400 MSM in San Francisco, Atlanta, and Boston, with 200 enrolled at the San Francisco site and is described in detail in published reports elsewhere [32]. The randomized trial checklist is provided in S1 File and the protocol is provided in S2 File. Participants were randomized in four equal proportions to immediate or delayed receipt of daily TDF or placebo, by the study statistician using a permuted block randomization scheme (blocks of 8 with 2 assignments in each of the four arms). Recruitment for the study occurred between January 2005 and January 2007. Two year follow-up was completed at all sites by July 2009, prior to the availability of any PrEP trial efficacy results [1–6]. All San Francisco site participants who attended a study visit between October 2007 and June 2008 and had been on study drug (either TDF or placebo) for ≥ 3 months were invited to participate in the sub-study. The primary inclusion criteria were male sex at birth, 18–60 years of age, HIV-1 negative by lab confirmation, hepatitis B surface antigen negative by lab confirmation, normal urinalysis and serum electrolytes and report of anal sex with a male partner in the past 12 months. Primary exclusion criteria include those with a history of chronic renal disease or concurrent use of nephrotoxic medications or HIV antiretrovirals.

#### Ethical considerations

Written informed consent was obtained separately for the parent study and sub-study from each study participant. All procedures performed in studies involving human participants were in accordance with the ethical standards of the institutional and/or national research committee and with the 1964 Helsinki declaration and its later amendments or comparable ethical standards. Both the parent study and this sub-study received approval from the University of California San Francisco Committee on Human Research (CHR #10–04109, initial approval on November 2, 2004).

### Sample collection and processing

Hair was collected at the sub-study enrollment visit. Viable PBMCs collected as part of the parent protocol every 6 months were selected for testing at either the same visit as hair collection if available (33% of samples) or at the nearest neighboring quarterly visit (either 3 months before or after hair collection) (67% of samples). Plasma samples collected quarterly per the parent protocol were selected for testing to match both hair and PBMC collection dates. Timing of collection of plasma samples in relation to last dose was not specified per protocol and varied by subject. All samples were collected and tested by staff who were blinded to study arm assignment. In plasma and hair specimens, TFV was measured and for PBMCs, TFV-diphosphate (TFV-DP) was measured, using the methods outlined for all three biomatrices below. TFV undergoes intracellular phosphorylation in PBMCs, ultimately resulting in TFV-DP which has a substantially longer half-life than TFV [33].

#### Plasma

Plasma was collected in EDTA tubes and analyzed for TFV via liquid chromatography/tandem mass spectrometry (LC/MS/MS) [34]. The TFV plasma assay was validated from 10 ng/mL to 1000 ng/mL. The lower limit of quantification (LLOQ) for TFV was 10 ng/mL. TFV in the quantifiable range would represent a dose within 48–72 hours.

#### PBMC

Procedures for processing viable PBMCs have been previously described [11]. A validated LC/MS/MS assay analyzed PBMC TFV-DP [35] with a linear range of 2.5 to 2000 femtomole (fmol)/sample. Approximately 1 to 2 million viable cells were typically extracted (representing the sample) and results reported as fmol/million viable cells. The LLOQ for TFV-DP was 2.5 fmol/sample. TFV-DP accumulates with dosing. In a prior PK study involving directly observed dosing of different dosing frequencies of TDF (STRAND), the median (IQR) TFV-DP concentration for 2 doses per week was 11 fmol/10^6^ cells (6 to 13 fmol/10^6^ cells), four doses per week was 32 fmol/10^6^ cells (25 to 39 fmol/10^6^ cells) and seven doses per week was 42 fmol/10^6^ cells (31 to 47 fmol/10^6^ cells) [11] after 6 weeks.

#### Hair

Approximately 100 strands of hair were cut as close as possible to the scalp in the occipital region and the distal portion was labeled [36, 37]. The proximal section of the hair sample (approximately 1 cm, reflecting the last month of dosing) was chopped to ~1–2 mm length segments with scissors and weighed, processed and analyzed using LC/MS/MS. The TFV in the cut hair sample was extracted with 50% methanol/water containing 1% trifluroacetic acid and internal standard (TFV-d6) in a 37°C shaking water bath overnight (>12 hours) and then analyzed by an LC/MS/MS method [38]. The relative error (%) and precision (coefficients of variation (CV)) for spiked quality control hair samples at low, medium and high concentrations were all <15%. These assays have been validated from 0.01 ng/mg to 0.400 ng/mg hair [38]. TFV accumulates significantly in hair. In a prior analysis after 6 weeks of dosing for each of 3 regimens in STRAND, the median (IQR) hair concentration for 2 doses per week was 0.012 ng/mg (0.008 to 0.021 ng/mg), four doses per week was 0.023 ng/mg (0.011 to 0.042 ng/mg) and seven doses per week was 0.038 ng/mg (0.021 to 0.053 ng/mg) [37]. The TFV assay in hair has been peer reviewed and approved by the Division of AIDS’ Clinical Pharmacology and Quality Assurance (CPQA) program.

### Non-biological measures of drug exposure

A medication event monitoring system (MEMS, Aardex) was used to determine the number of pill bottle cap openings occurring over 30 and 90 days. Self-reported regimen adherence was determined by audio computer-assisted self-interview (ACASI) using a visual analog scale (VAS) representing the percentage of pills taken in the last month. Announced pill counts were performed by study staff in the clinic at each visit.

### Statistical analysis

#### Outcome

The primary outcomes in the sub-study were log transformed TFV concentrations in plasma and hair, and log transformed TFV-DP concentrations in PBMCs.

#### Explanatory variables

Drug detection was first evaluated by receipt of active TDF or placebo. Biological and adherence-related factors influencing drug concentrations were then assessed, including age, weight, creatinine clearance, pill count, MEMS 30 and 90-day medication bottle openings, and self-reported adherence using a 10-point visual analog scale (VAS) assessed via ACASI. Additionally, self-reported hair color (categorized as reddish/blonde versus black/brown) and a self-reported history of cosmetic hair treatment (bleaching, highlighting or other) within 3 months preceding hair collection were assessed as predictors of hair TFV concentrations. Full gray hair was not included in hair color analyses due to the small number of individuals.

#### Analysis

Descriptive statistics were used to summarize baseline characteristics of sub-study participants, using the Kruskal-Wallis test for continuous variables and the Fisher exact test for nominal variables. We then calculated the proportions of participants assigned to active TDF or placebo with detectable levels in hair, plasma, and PBMCs. In-depth case reviews were performed for participants with drug detection results inconsistent with treatment assignment. The associations of age, weight, creatinine clearance, adherence measures, and hair color and treatment with log-transformed TFV or TFV-DP concentrations in hair, plasma and PBMCs in active arm participants were estimated using univariate linear regression models. Subsequently, individual multivariable linear regression models were used to measure the association of weight, CrCl, pill count, MEMS, and self-report with the three separate biological measures (plasma, PBMC, and hair). Coefficients obtained through regression modeling were then back-transformed to be interpretable as percentage changes. Confounders were considered for each model and noted in the results. The addition of a small constant (+1x10^-6^ ng/mg) to tenofovir plasma and PBMC values prior to log transformation reduced the importance of differences between very low levels, while preserving the approximate interpretation of back-transformed regression coefficients as percent changes in drug concentration. This allows all values, including those below the LLOQ, to be included in the analysis. Spearman correlation coefficients between various pharmacologic measures of TFV and non-pharmacologic measures of adherence were calculated in active arm participants only, using samples obtained concurrently or at the nearest visit (for some PBMCs—see sample collection and processing above). Concurrent refers to obtaining two biological specimens at the same visit. Finally, in order to understand the impact of outliers on results, linear regression models were re-run restricting to ≥60% MEMS adherence, representing those with adherence that may be sufficient for a protective effect. Analyses were performed using Stata Version 13.2 (Stata Corp, College Station, TX). Figures were generated in using Stata and R (version 3.2.3, Vienna, Austria).

## Results

**Fig 1:**
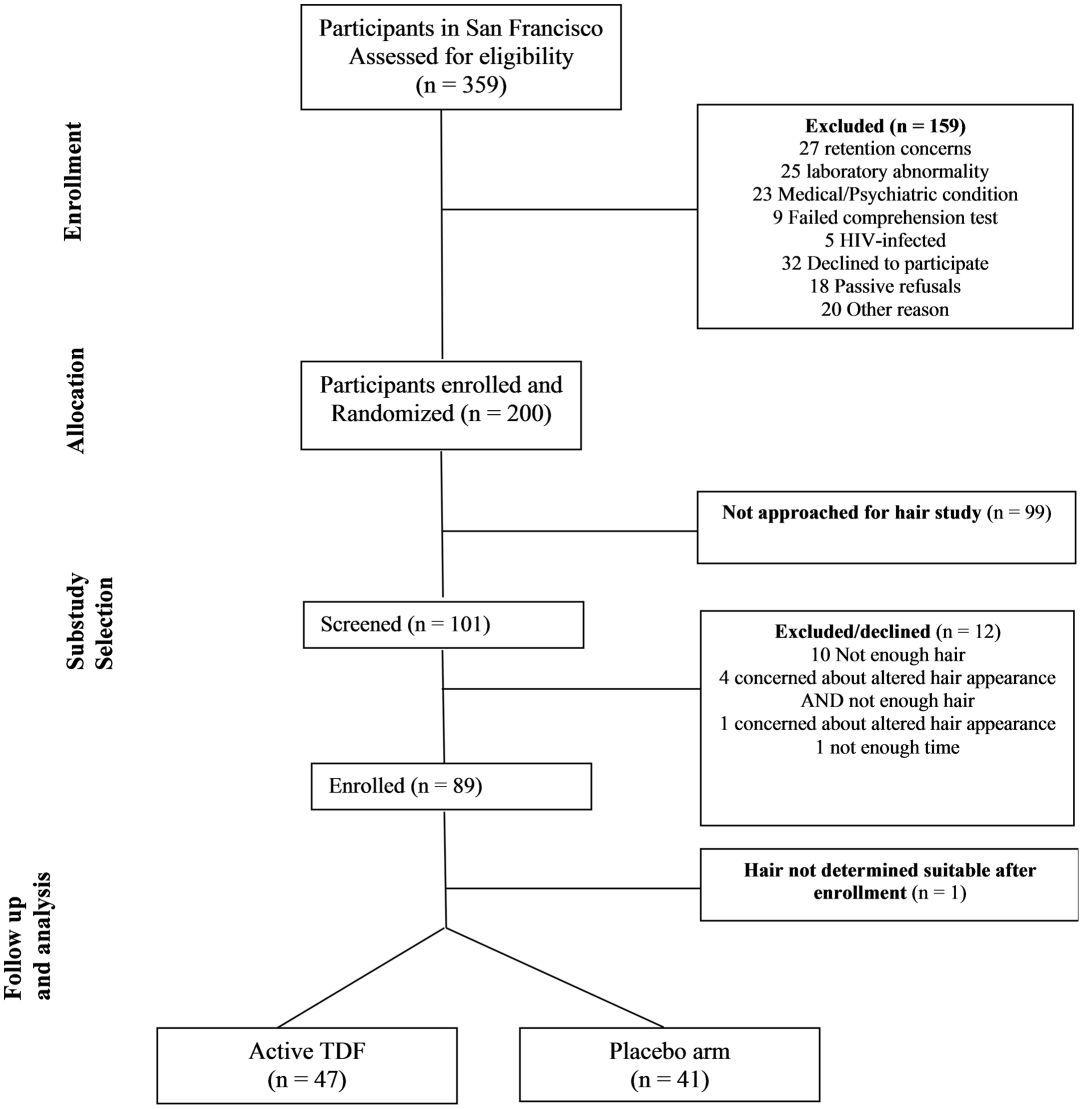
Study design and participant disposition.

In total, 101 men of the 200 enrolled at the San Francisco site were eligible and screened for participation in this sub-study and 88 (88%) provided written informed consent to participate; all 88 men contributed data to this analysis (Fig 1). Table 1 shows the baseline characteristics of these participants in the active and placebo study arms. These 88 participants were compared to the remaining 112 who were enrolled at the San Francisco site, but not included in this sub-study and no statistically significant differences were found with respect to race, age, creatinine clearance or weight (by Kruskal-Wallis one-way test of variance for all except race which was evaluated using Fisher’s exact test). This population was mostly of White race and middle aged (median age of 41 years). Demographic characteristics were similar across arms, although there was weak evidence suggesting that participants in the placebo arm demonstrated lower adherence as assessed by 30 day MEMS medication bottle-openings compared to those in the active arm (median 77% versus 87%, respectively, p = 0.09 by Kruskal-Wallis one-way test of variance). In participants randomized to the active arm, median (range) TFV or TFV-DP concentrations in hair, plasma and PBMC were 0.05 ng/mg (<0.01–0.21 ng/mg), 83 ng/mL (<10–367 ng/mL) and 40 fmol/million cells (<5–102 fmol/million cells), respectively. Overall, 81% of participants had TFV hair concentrations consistent with high levels of protection (≥0.023 ng/mg, consistent with 4 or more doses/week) [11, 37].

**Table 1:** Baseline characteristics of participants in the active and placebo participant arms of the US CDC PrEP safety study.

Characteristic	Active TDF (n = 47)	Placebo arm (n = 41)	Total (n = 88)
Age, median (IQR)	40 (35, 45)	45 (33, 52)	42 (34, 49)
Race, N (%)			
*White*	40 (85.1)	40 (85.1)	73 (83.0)
*Black*	2 (4.3)	0 (0.0)	2 (2.3)
*Asian/Pacific Islander*	4 (8.5)	2 (4.9)	6 (6.8)
*Other*	1 (2.1)	6 (14.6)	7 (8.0)
Creatinine clearance, median in ml/min (IQR)	122 (97, 144)	118 (102, 134)	122 (102, 141)
Weight, median in kg (IQR)	86 (77, 99)	83 (76, 94)	84 (77, 98)
Hair color, N (%)			
*Black/Brown*	36 (76.6)	32 (78.1)	68 (77.3)
*Reddish/Blonde*	9 (19.2)	7 (17.1)	16 (18.2)
*Totally gray*	2 (4.3)	2 (4.9)	4 (4.6)
Hair Treatment, N (%)			
*Any*	9 (19.2)	7 (17.1)	16. (18.2)
*Bleaching*	1 (2.1)	0 (0.0)	1 (1.1)
*Highlighting*	2 (4.3)	2 (4.9)	4 (4.6)
*None*	35 (74.5)	32 (78.0)	67 (76.1)
Adherence, median % (IQR)			
*Pill counts*	90 (80, 97)	91 (76, 97)	91 (77, 97)
*MEMS (30-day adherence)*	87 (77, 93)	77 (60, 93)	85 (67, 93)
*ACASI self-report by VAS (30 days)*	90 (90, 90)	90 (90, 90)	90 (90, 90)

TDF = tenofovir disoproxil fumarate; IQR = interquartile range; MEMS = medication electronic monitoring system; ACASI = audio computer-assisted self-interview; VAS = visual analog scale

**Fig 2:**
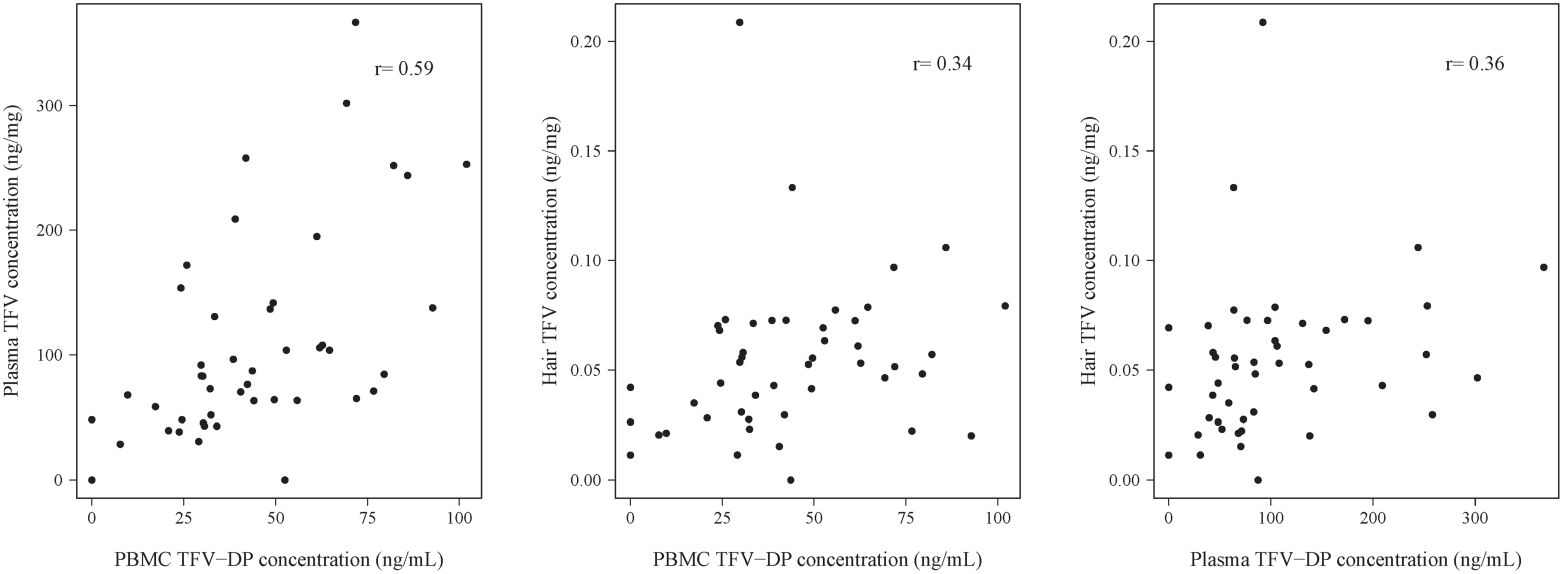
Scatterplots of PBMC TFV-DP concentration versus plasma TFV concentration (2a), PBMC TFV-DP concentration versus hair TFV concentration (2b) and plasma TFV concentration versus hair TFV concentration (2c).

Drug was detected in plasma, PBMCs, and hair in almost all active arm participants, and not detected in almost all placebo arm participants (Table 2). One participant randomized to the placebo arm had detectable drug concentrations in all three biomatrices collected and measured on the same study day. When hair and plasma samples were obtained concurrently, 46/47 (97.9%) active arm participants had detectable TFV in both matrices. When PBMCs and plasma samples were obtained concurrently, 44/47 (93.6%) active arm participants had detectable TFV-DP and TFV, respectively. Table 3 shows Spearman correlation coefficients for each measure of adherence or drug exposure against all other measures in the study and Fig 2 shows the scatterplots for the biological measures. Spearman correlation coefficients ranged between 0.34 and 0.59 for pharmacologic measures, and was highest for plasma and PMBCs. In general, the non-biological measures (self-report using VAS measured by ACASI, announced pill count, and MEMS) were moderately correlated with each other. Self-reported adherence using VAS measured by ACASI was poorly correlated with all the drug level measures. MEMS and announced pill count adherence data were modestly correlated with hair and plasma levels.

**Table 2:** Detection of tenofovir in hair, plasma and peripheral blood mononuclear cells in active and placebo participant arms in the US CDC PrEP safety study.

	TFV Detectable in Hair		TFV Detectable in Plasma matched to hair		TFV Detectable in Plasma matched to PBMCs		TFV Detectable in PBMCs	
	No	Yes	No	Yes	No	Yes	No	Yes
Treatment	1	46	1	46	3	44	3	44
Placebo	40	1*	40	1*	10	1*	10	1*

TFV = tenofovir; PBMC = peripheral blood mononuclear cells;

*tenofovir detected in the same individual, on the same date, in the placebo arm across all three biomatrices

**Table 3:** Spearman correlation coefficients between various measures of TDF exposure in active arm participants only (n = 47) in the US CDC PrEP safety study.

	Self-report	Pill count*	MEMS*	PBMC TFV-DP	Plasma TFV	Hair TFV
Self-report	1.0					
Pill count	0.44	1.0				
MEMS	0.32	0.52	1.0			
PBMC TFV-DP	0.06	0.17	0.11	1.0		
Plasma TFV	0.06	0.36	0.37	0.59	1.0	
Hair TFV	0.06	0.38	0.20	0.34	0.36	1.0

*MEMS and Pill count data are for 90 days before either PBMC, plasma, or hair collection

Table 4 provides further detail on the five participants in the active arm who had undetectable drug in at least one specimen type. TFV was detected in hair from four of these men, with undetectable drug in both plasma and PBMCs in two individuals, and drug detected in plasma or PBMCs in one individual each. One participant with an undetectable TFV concentration in hair had drug detected in the other two matrices. The patterns of drug levels and how they relate to the non-biological measures, as well as possible interpretations of the various patterns, are provided in Table 4.

**Table 4:** Pharmacokinetic and non-pharmacokinetic adherence measures among five participants who had at least one sample with undetectable drug in the US CDC PrEP safety study.

	Detectable tenofovir in biological measures of drug exposure					Count data for non-biological measures of drug exposure					Most likely Interpretation
Participant	Hair (level ng/mg hair)	Plasma on hair collection date (ng/mL)	Plasma on PBMC collection date (ng/mL)	PBMC on hair collection date (fmol/sample)	PBMC if done on date other than hair	MEMS openings 90 days before hair (%)	MEMS openings 90 days before PBMC (%)	Self-report by ACASI using VAS (%)	Pill counts in 90 days before hair	Pill counts in 90 days before PBMC	-
1	Yes (0.026)	Yes (48.3)	PBMCs, plasma and hair collected on same date	No (BLQ)	n/a	92.2	92.2	90	95.5	95.5	False negative PBMC result or remote dosing (suggested by hair) with dosing holiday (PBMC BLQ) and then very recent dosing before clinic visit (plasma elevated)
2	Yes (0.042)	Yes (99.5)	No (BLQ)	n/a	No (BLQ) (58 days before hair)	68.9	1.1	100	73.3	N/A	Low/no adherence before PBMC collection date, but higher adherence before hair collection date
3	Yes (0.069)	Yes (446)	No (BLQ)	n/a	Yes (52.48 fmol/sample, 103 days after hair)	34.4	31.1	90	86.0	38.8	Adherence throughout the duration with waning adherence in the last few days prior to PBMC sampling, or false negative plasma test.
4	No (BLQ)	Yes (188)	Yes (87.4)	n/a	Yes (43.62 fmol/sample, 91 days after hair)	42.2	75.6	90	82.4	80.2	False negative hair result, or, less likely, recent adherence without remote adherence.
5	Yes (0.011)	No (BLQ)	No (BLQ)	n/a	No (BLQ) (32 days after hair)	0	0	70	1.6	21.9	Remote adherence without recent adherence.

PBMC = peripheral blood mononuclear cells, MEMS = medication electronic monitoring system; ACASI = audio computer-assisted self-interview; VAS = visual analog scale; BLQ = below lower limit of quantification

Factors associated with tenofovir levels in hair, plasma and PBMCs are shown in Table 5. Reddish/blonde hair color (when compared to black/brown hair, *p* = 0.04) and self-report of any cosmetic hair treatments in the last 3 months (*p* = 0.016) were associated with lower TFV level among participants randomized to the TFV arm in univariate analyses, but these associations were not maintained in multivariate models. We did not find evidence for interaction between these two factors (*p* = 0.60). While older age was associated with increasing plasma concentrations, weight and creatinine clearance were not associated with concentration of study drug in any of the three specimens in univariate or multivariable analyses, although normal creatinine clearance was an eligibility criterion for the overall trial, and thus variability in this characteristic was limited.

**Table 5:** Percent differences in tenofovir in hair, plasma and peripheral blood mononuclear cells in active arm participants, by linear regression (univariate and multivariable results presented), in the US CDC PrEP safety study.

	Hair, % difference (95% CI)		Plasma, % difference (95% CI)		PBMC, % difference (95% CI)	
Explanatory variable	Unadjusted	Adjusted	Unadjusted	Adjusted	Unadjusted	Adjusted
Age (per 10 years)	20 (-3, 44), *p* = 0.09	-	75 (23, 128), ***p* = 0.005**	-	34 (-11, 79), *p* = 0.14	-
Weight (per 10 kg)	-5 (-16, 7), *p* = 0.41	*-8 (-18*, *3)*, *p = 0*.17*	8 (-13, 29), *p* = 0.47	*1 (-17*, *18)*, *p = 0*.94*	2 (-19, 23), *p* = 0.87	*-2 (-23*, *18)*, *p = 0*.81*
CrCl (per 10 mL/min/1.73 m^2^)	-6 (-12, 1), *p* = 0.08	*-2 (-13*, *9)*, *p = 0*.75**	-7 (-17, 4), *p* = 0.22	*-1 (-18*, *16)*, *p = 0*.93**	-4 (-15, 8), *p* = 0.52	*2 (-18*, *22)*, *p = 0*.84**
Pill count (per 10%)	12 (4, 21), ***p* = 0.003**	*12 (4*, *20)*, *p = 0*.005**	24 (9, 38), ***p* = 0.002**	*18 (5*, *32)*, *p = 0*.008**	17 (2, 32), ***p* = 0.03**	*14 (-2*, *30)*, *p = 0*.08**
MEMS 30-day (per 10%)	2 (-5, 9), *p* = 0.52	*2 (-5*, *9)*, *p = 0*.50**	15 (3, 27), ***p* = 0.012**	*12 (1*, *23)*, *p = 0*.03**	13 (2, 24), ***p* = 0.02**	*12 (1*, *23)*, *p = 0*.03**
ACASI self-report by VAS (per 10%)	6 (-12, 25), *p* = 0.50	*5 (-13*, *24)*, *p = 0*.59**	34 (-3, 71), *p* = 0.07	*23 (-9*, *55)*, *p = 0*.15**	17 (-18, 53), *p* = 0.34	*13 (-22*, *48)*, *p = 0*.48**
Reddish/blonde hair color (vs black/brown)	-32 (-62, -2), ***p* = 0.04**	-	-	-	-	-
Any hair treatment^Δ^	-34 (-63, -4), ***p* = 0.025**	-	-	-	-	-

CI = confidence interval; CrCl = creatinine clearance; PBMC = peripheral blood mononuclear cells; TFV = tenofovir; MEMS = medication electronic monitoring system; ACASI = audio computer-assisted self-interview; VAS = visual analog scale

unadjusted refers to univariate regression modeling; adjusted refers to multivariate modeling for the variable in a given row

* for age only or the variable in a given row

** for age and weight;

^Δ^ excludes full gray hair

Average plasma TFV and PBMC TFV-DP concentrations substantially increased (15% and 13%, respectively) with each 10% increase in MEMS 30-day adherence by medication bottle -openings, although the effects for both were no longer statistically significant among participants with ≥ 60% adherence by MEMS, suggesting the overall association was driven by the few participants with low adherence on MEMS. Announced pill counts were the only non-pharmacologic adherence measure statistically significantly associated with hair (*p* = 0.003), plasma (*p* = 0.002) and PBMC (*p* = 0.03) concentrations, but again the effect was diminished when limiting results to those with ≥ 60% adherence as assessed by MEMS. Restricting to ≥ 60% adherence resulted in the following proportions removed for each statistical test, reported as test [number removed from analysis (% removed of total)]: impact of MEMS on hair concentration [5 (11%)], impact of MEMS on plasma concentration [5 (11%)], impact of MEMS on PBMC concentration [9 (19%)], impact of pill count on hair concentration [5 (12%)], impact of pill count on plasma concentration [5 (12%)], impact of pill count on PBMC concentration [4 (10%)]. These findings did not change substantially in multivariable analyses adjusting for age and weight. Self-reported adherence by VAS measured by ACASI was not a statistically significant predictor of TFV or TFV-DP in hair, plasma or PBMC in univariate or multivariable models.

## Discussion

In this sub-study of San Francisco site participants of the CDC-sponsored safety study of TDF PrEP, uptake of hair collection was found to be high and detection of study drug in hair, plasma, and PBMCs was highly concordant with treatment arm assignment. Study drug was detected in almost all participants randomized to the active TDF arm, and appropriately not detected in all but one participant randomized to placebo. This latter individual was possibly a surreptitious PrEP user. Drug concentrations in hair, plasma, and PBMCs were less strongly correlated with each other than in other studies [15], and at best moderately correlated with adherence as measured by MEMS medication bottle openings and pill counts. Drug concentrations were poorly correlated with self-reported adherence using VAS measured by ACASI.

Reasons for the relatively weak correlations among all of the measured drug concentrations are unclear. Notably, most participants had drug concentrations suggesting high levels of adherence [11, 37], so that biological and analytical variability likely represents a larger proportion of overall observed variability. Additional reasons for relatively weak correlations would include non-concurrent measurements, variability in use over time, genetic factors that may impact drug measurement in one setting versus another or limitations of the current assays. The lack of substantial association between self-reported adherence and drug concentrations in any of the three biological specimens is consistent with a recent analysis in other PrEP trials by our group [15] and consistent with findings from other PrEP trials [9, 10, 15, 39]. MEMS medication bottle openings were associated with plasma and PBMC drug concentrations, but the association was weaker among the participants with MEMS-measured adherence of ≥ 60%. In contrast to findings in intermittent PrEP trials in Kenya and Uganda [15], MEMS medication bottle-openings were not substantially associated with concentration of tenofovir measured in hair in this study. Interestingly, announced pill counts were associated with TFV drug concentration measured in hair and plasma, and TFV-DP drug concentration measured in PBMCs, but again the correlation was weaker when restricting analyses to those with higher MEMS adherence. Finally, hair color and coloring or chemical treatment were associated with lower overall concentrations of TFV in hair in univariate, but not multivariable, modeling.

These findings highlight the variability across adherence measures which may reflect imprecision in measurement, differences in time period of assessment of different measures, social-desirability bias for non-pharmacologic measures, and biological and analytic variability for PK measures. Accounting for variability between adherence measures may improve interpretation and use of adherence measures in future PrEP trials. The high concordance of PK drug detection and treatment arm assignment in this study, along with the strong relationship observed between dose and drug levels in hair and PBMCs in a PK study using directly observed dosing [37], support the use of objective measures of drug exposure where possible. Self-reported adherence can be subject to over-reporting bias [9, 10, 15, 39] and this report further supports that the discordance between biological measures and self-report of adherence using VAS via ACASI may be due to the inaccuracy of the latter. MEMS medication bottle-openings may be a more accurate approximation of adherence than self-report [15, 40] and can provide data on patterns of adherence, but cannot measure ingestion of drug. Pill counts in HIV prevention trials have proven useful [16, 17], but they may be limited by pill dumping or sequestering [9, 10, 39] and a lack of method standardization across studies [18]. In our study, announced pill counts and MEMS as indicators of dosing were moderately correlated with each other and provided a more consistent assessment of tenofovir concentration compared with self-report using VAS measured by ACASI when evaluated against the biological measures of drug exposure. Biological measures can confirm actual ingestion of study drug and have been used extensively in PrEP studies, both as an indicator of adherence to study product and as a correlate of antiviral protection [1, 9–12, 15, 30, 41]. Strong correlation between measures in different biomatrices in the PrEP setting has been previously reported [15].

Discrepancies in drug detection in the different matrices may provide information on patterns of dosing, but false negative or positive tests should also be considered. The various half-lives of TFV in plasma and hair and of TFV-DP in PBMCs are helpful in understanding the proximity of drug consumption with respect to measurement of drug level, as well as the accumulation of doses for hair and PBMC. For example, participant #3 had detectable drug in hair and PBMC, but not plasma, consistent with recently declining adherence. Participants #2 and #5 also had detectable hair levels, but they both had undetectable PBMC TFV-DP concentrations in addition to undetectable TFV in plasma, which may represent more remote dosing.

While the optimal strategy and frequency of drug monitoring for PrEP has yet to be defined, cost and feasibility of specimen collection and processing are important considerations, particularly when considering PrEP adherence measurement during wide-scale PrEP implementation and scale-up, including in low resource settings. Current PK measures of adherence all require LC/MS-MS, which involves expensive equipment, and, except for hair, require biohazard precautions for handling, processing and storing specimens. Dried blood spots (DBS) are easier to collect and process than PBMCs, but DBS still require storage in freezers and require standardization when the hematocrit falls outside the normal range [42]. Lower-cost methods to assay pharmacologic exposure measures, with potential point-of-care testing, are currently under development, including in hair, urine and DBS [43–45]. Understanding how these measures may be combined to most accurately ascertain patterns of adherence and drug exposure, in ways that are affordable, feasible and informative, is an important research priority.

There were a number of strengths to this study. We obtained multiple measures of drug adherence and exposure, allowing comparisons of three biological measures and three commonly used non-biological measures. Specificity of the biological measures could be estimated among participants randomized to placebo (via MEMs, VAS using ACASI, and pill count), in contrast to a previously published analysis of two PrEP trials which compared biological measures of drug exposure and non-biological measures of drug adherence in active arm participants only and excluded those randomized to placebo [15]. This is important given that placebo-controlled PrEP trials are not ethical going forward and therefore this study provides an important insight into how well these measures identify true negatives. There are also some limitations to the interpretation of these data. This study only evaluated adherence measures at a single time-point and did not provide information on the measurement of longitudinal patterns of adherence. The lack of a true gold standard in the field of adherence measurement in HIV-negative persons is another limitation. We had to use treatment assignment as a proxy, but were able to interpret the three biologic and three non-biologic measures for the six participants whose results were not clearly consistent with treatment assignment. The drug concentrations were not assessed in relationship to the primary outcome of interest, specifically HIV acquisition, given the relatively small number of participants and relative rarity of this outcome in the PrEP trial setting. Also, the acceptability and performance of different indicators of adherence may be different in the context of a randomized clinical trial compared with clinical practice. Although there was low racial and gender diversity in this study, the hair assay has been validated in diverse settings with a variety of antiretroviral agents [15, 29, 36, 37, 44, 46, 47]. Finally, assuming that the variability in drug exposure is only a function of variability of adherence may be inaccurate because individual pharmacokinetics also determine drug exposure and may be influenced by genetic variability and other factors. Recent data have found genetic variants that impact tenofovir pharmacokinetics, and such variability may be dependent on what biological tissue is sampled [48].

## Conclusions

In conclusion, in a randomized placebo controlled trial of tenofovir-based PrEP in MSM, concentrations of TFV or TFV-DP in hair, plasma and PBMCs were detected in very high proportions in the active arm; MEMS medication bottle-openings and pill counts were modestly associated with biological measures of drug exposure; and self-report using VAS measured by ACASI correlated poorly with drug exposure. While self-reported measures often overestimate adherence, they may provide important information about patterns of PrEP use and the client’s goals for PrEP use, which cannot be obtained using biological measures. Such biological measures can assist in identifying other self-reported measures which are less subject to bias and play an important role in objectively measuring drug exposure in PrEP research and implementation.

## Supporting information

S1 File Specific checklist items for randomized trials. (DOC)

S2 File Specific protocol information for the randomized trial for this report. (DOC)

S1 Data Provides the minimally sufficient data to reproduce the output. (XLSX)
